# *Lycium barbarum* polysaccharides restore adverse structural remodelling and cardiac contractile dysfunction induced by overexpression of microRNA‐1

**DOI:** 10.1111/jcmm.13740

**Published:** 2018-08-17

**Authors:** Rong Zhang, Yi Xu, Huifang Niu, Ting Tao, Tao Ban, Linyao Zheng, Jing Ai

**Affiliations:** ^1^ Department of Pharmacology (State‐Province Key Laboratories of Biomedicine‐Pharmaceutics of China, Key Laboratory of Cardiovascular Medicine Research, Ministry of Education) College of Pharmacy Harbin Medical University Harbin China

**Keywords:** calmodulin, cardiac function, cardiac myosin light chain kinase, *Lycium barbarum* polysaccharides, microRNA‐1, structural remodelling

## Abstract

MicroRNA‐1 (miR‐1) stands out as the most prominent microRNA (miRNA) in regulating cardiac function and has been perceived as a new potential therapeutic target. *Lycium barbarum* polysaccharides (LBPs) are major active constituents of the traditional Chinese medicine based on *L. barbarum*. The purpose of this study was to exploit the cardioprotective effect and molecular mechanism of LBPs underlying heart failure. We found that LBPs significantly reduced the expression of myocardial miR‐1. LBPs improved the abnormal ECG and indexes of cardiac functions in P‐V loop detection in transgenic (Tg) mice with miR‐1 overexpression. LBPs recovered morphological changes in sarcomeric assembly, intercalated disc and gap junction. LBPs reversed the reductions of CaM and cMLCK, the proteins targeted by miR‐1. Similar trends were also obtained in their downstream effectors including the phosphorylation of MLC2v and both total level and phosphorylation of CaMKII and cMyBP‐C. Collectively, LBPs restored adverse structural remodelling and improved cardiac contractile dysfunction induced by overexpression of miR‐1. One of the plausible mechanisms was that LBPs down‐regulated miR‐1 expression and consequently reversed miR‐1‐induced repression of target proteins relevant to myocardial contractibility. LBPs could serve as a new, at least a very useful adjunctive, candidate for prevention and therapy of heart failure.

## 1 INTRODUCTION

Heart failure (HF) is a common, disabling and potentially deadly condition and remains the only cardiovascular disease with an increasing hospitalization burden and an ongoing drain on health care expenditures, despite several therapeutic approaches have reduced cardiovascular morbidity and mortality.^1^, ^2^ Therefore, to explore the pathogenesis of such disease and look for novel effective drugs with low side‐effects would have great impacts on its prevention and clinical management of HF. Recently, constituents from natural herbs have attracted attention with regard to pharmaceutical development. MicroRNA (miRNA), a small non‐coding RNA, is emerged as a critical node in post‐transcriptional regulation and a newly discovered class of gene regulatory factors at the core of human physiology and disease.^3^, ^4^ Increasing lines of evidence have been rapidly evolving for the crucial roles of miRNAs in regulating diverse aspects of cardiac function and progression of HF.^4^, ^5^ And notably microRNA‐1 (miR‐1), a cardiac‐enriched miRNA, is in most close relation to heart conditions, and the changes in its expression have been discovered in a variety of heart diseases.^6^, ^7^, ^8^, ^9^, ^10^ Our previous report revealed that outcomes of miR‐1 overexpression induced adverse structural remodelling, which impaired cardiac contractile and diastolic function and even caused HF.^11^ MiR‐1 could result in an epigenetic defect as cardiac hypertrophy by microinjecting fragments of miR‐1.^12^ The functional significance of the findings uncovered that miR‐1 might be a trigger and sustainer for the structural remodelling and dysfunction of HF. Down‐regulation of miR‐1 may produce cardioprotective effects^7^, ^8^, ^13^ and miR‐1 has been perceived as a new therapeutic target for heart diseases. Therefore, it is valuable to develop new candidates regulating miR‐1 by which to treat relevant heart diseases.

*Lycium barbarum* polysaccharides (LBPs) are important active constituents extracted from the traditional Chinese herb *L. barbarum*. There are scientific proofs of its pharmacological and biological functions including anti‐oxidative properties,^14^ immunomodulation,^15^ antitumor activity,^16^ anti‐ageing effect,^17^ neuroprotection,^18^ hypoglycaemic and hypolipidemic effects^19^ and male fertility‐facilitating,^20^ indicating extensive application prospects on relevant diseases. To the best of our knowledge, there are only a few studies concerning the effect of LBPs on cardiovascular system. Electrocardiographic and biochemical evidence were found that LBPs elicit a typical cardioprotective effect against Doxorubicin‐related oxidative stress in rats and dogs.^21^, ^22^ LBPs reduced myocardial apoptosis and injury in ischemia/reperfusion process of rat heart and could prevent the development of cardiovascular diseases.^23^, ^24^ However, until now, no available information has addressed the effects of LBPs on cardiac structure and function in HF. Given its biological property, it is conceivable that LBPs may produce beneficial actions in preventing the development of HF and act as a potential therapy option. This study was aimed to investigate the effects of LBPs on impaired cardiac function and structural remodelling induced by overexpression of miR‐1 and to unravel the underlying molecular mechanism for exploiting therapeutic potential on HF.

## 2 MATERIALS AND METHODS

### 2.1 Preparation of *Lycium barbarum* polysaccharides

*Lycium barbarum* polysaccharides were prepared using the extraction procedure optimized by Jingcheng Tang.^25^ Briefly, the dry fruits of *L. barbarum* (0.5 kg, purchased from Xi'an Tianyuan Biologics Plant, China) were immersed in deionized water for 12 hours, boiled for 1.5 hours and extracted twice. The combined aqueous extracts were concentrated in vacuum and precipitated by 95% ethanol for 48 hours at room temperature. The precipitate was filtered at reduced pressure and dried by lyophilization. We got a yield of 37.8% LBPs powder. The crude LBPs have been deproteinated by the Sevag method, and intensively dialysed for 2 days against distilled water (cut‐off Mw 3500 Da). The solution of LBPs samples were scanned under UV‐VIS spectrometer in the range from 190 to 400 nm, and there was no absorbance peak at 280 and 260 nm, implying that the protein and nucleic acid were absent in this polysaccharide. LBPs were diluted by ddH_2_O in our study.

### 2.2 Generation of miR‐1 transgenic (Tg) mice

All experimental procedures and protocols used in this investigation received approval by the Institutional Animal Care and Use Committee of Harbin Medical University, which conforms to the Guide for the Care and Use of Laboratory Animals published by the US National Institutes of Health (NIH Publication No. 85‐23, revised 1985). We generated Tg mouse line for cardiac‐specific overexpression of miR‐1 driven by the α‐myosin heavy chain (α‐MHC) promoter as previously reported.^11^ Briefly, sexually immature female C57BL/6 mice (4‐5 weeks of age) were applied to obtain sufficient quantity of eggs (>250) for microinjection. The mice used in this study were the fifth generation or later.

### 2.3 Administration of LBPs to mice

Animals were divided into three groups: C57BL/6 male wild‐type (WT) littermate mice, miR‐1 Tg mice, miR‐1 Tg mice received LBPs. LBPs were delivered into miR‐1 Tg mice at 3 months of age through intragastric administration at a dosage of 200 mg/kg/d^22^, ^26^ for 1 month. The age‐matched WT mice and miR‐1 Tg mice were received 0.9% NaCl (0.2 mL/d) as the vehicle. In the next 2 months, LBPs was mixed in water and administered to the mice by drink at the same dose. Monitoring of drinking quantity manifested no change in all mice. Measurements were made when the mice were at an age of 6 months.

### 2.4 Culture of neonatal rat ventricular cardiomyocytes (NRVCs)

Hearts from 1‐ to 3‐day‐old Wistar rats were excised, and the ventricular myocardium was minced in Dulbecco's modified Eagle's medium (DMEM, Invitrogen) and cells were dissociated with 0.25% trypsin‐EDTA solution (Beyotime, China). After centrifugation, the collected isolated cells were plated onto 25 cm^2^ cell culture flask (Corning Incorporated, USA) for 100 minutes to separate ventricular myocytes from the faster attaching non‐myocytes. The re‐collected cells were then seeded in a six‐well plate (2 × 10^5^/well) in DMEM containing 10% foetal bovine serum (FBS) and 0.1 mmol/L bromodeoxyuridine (sigma). Cells were used for experiments 48‐72 hours after isolation when demonstrating rhythmic contractions.

### 2.5 Administration of LBPs to NRVCs

NRVCs (2 × 10^5^/well) were incubated with 2 mL fresh FBS‐free medium in six‐well plates and administered incremental dose of LBPs (100, 400 and 800 μg/mL).^27^, ^28^ For miR‐1 overexpression treated groups, NRVCs were transfected with 2.5 μg miR‐1 or negative control (NC) siRNA with X‐treme GENE siRNA transfection reagent (Cat.#04476093001, Roche). LBPs were administered with miR‐1 at the same time. At 48 hours post‐transfection, cells were harvested for total RNA or protein extraction. MiR‐1 and NC were synthesized by Shanghai GenePharma Co., Ltd. The sequence of rno‐miR‐1 is 5′‐UGGAAUGUAAAGAAGUGUGUAU‐3′. The sequence of NC is 5′‐UUCUCCGAACGUGUCACGUTT‐3′.

### 2.6 Detection of heart function

Mice were anaesthetized with sodium pentobarbital (60 mg/kg, intraperitoneal). After recording ECG for 10 minutes, pressure‐volume (PV) loops (Scisense, Ontario, Canada) measurements were performed with a 1.2F mouse pressure‐volume catheter (Pressure‐Volume Control Unit FV896B) which was retrogradely inserted into the left ventricle cavity through the right carotid artery to measure baseline arterial pressure haemodynamics in the closed chest. All data were analysed with iWork Labscribe2 Data Recording and Analysis software. Baseline haemodynamic values were obtained by averaging 300 beats recorded during steady‐state periods. The main measured parameters included ejection fraction (EF), cardiac output (CO), end‐systolic pressure (ESP), end‐diastolic pressure (EDP), end‐systolic volume (ESV), end‐diastolic volume (EDV), maximum derivative of change in systolic pressure over time (dP/dt_max_) and maximum derivative of change in diastolic pressure over time (dP/dt_min_).

### 2.7 Evaluation of morphological remodelling

For transmission electron microscopy (TEM) detection, heart tissues from left ventricles were removed and immersed in stationary liquid (pH 7.3) containing 3% glutaraldehyde, and then fixed in 2% Osmic acid (OsO_4_). After gradient dehydration tissues were embedded in epon with propylene oxide as an intermediary solvent. Ultrathin sections were processed, mounted onto formvar‐coated slot grids, and stained with uranyl acetate and lead citrate. Images were captured by a Hitachi H‐7650 electron microscope (Hitachi, H‐7650, Tokyo, Japan). The quantitative analysis was to measure the lengths of damaged and total intercalated disc by Image J. The result was represented as the percentage of damaged length to the total. Haematoxylin and eosin (H&E) staining was performed by routine method. Hearts were taken and fixed in zinc formalin for 24‐48 hours then processed using a Sakura Tissue Tek VIP5 processor. Samples were embedded in paraffin and sectioned longitudinally from the identical plane of the initial portion of the ascending aorta at 4 μm using a microtome. Sections were stained with haematoxylin and eosin for identification. Heart‐to‐bodyweight ratio (HW/BW) was calculated for each group. The length of sarcomeres was evaluated by Image‐Pro Plus 6.0 (Media Cybernetics, Bethesda, MA).

### 2.8 Quantification of miR‐1 levels in Tg mice or NRVCs

The total RNA samples were isolated using Trizol and phenol/chloroform extraction procedures. MiR‐1 level was quantified by the TaqMan^®^ MicroRNA Reverse Transcription Kit (Cat.#4366596, Applied Biosystems) and the TaqMan^®^ MicroRNA Assay (for mice: target sequences: UGGAAUGUAAAGAAGUAUGUAU, Cat.#002222, Applied Biosystems; for rats: target sequences: UGGAAUGUAAAGAAGUGUGUAU, Cat.#002064, Applied Biosystems). U6 (Cat.#001973, Applied Biosystems) was used as an internal control. The quantitative Real‐time PCR (qRT‐PCR) was performed on 7500 FAST Real‐Time PCR System (Applied Biosystems) for 40 cycles.

### 2.9 Western blot analysis

The left ventricles of mice or cultured NRVCs were homogenized in lysis buffer (RIPA buffer 60%, SDS 40% and protease inhibitor cocktail 1%) on ice and then centrifuged at 18 000 ***g*** at 4°C for 30 minutes to remove the insoluble pellet. Protein concentration in the supernatant was determined by the BCA Protein Assay Kit (Bio‐Rad, Hercules, CA, USA). Equal amounts of protein (60 or 80 μg) were loaded on a 10% or 15% SDS‐PAGE gel. The lysates were resolved by electrophoresis (70 V for 30 minutes and 100 V for 1.5 hours) and transferred onto nitrocellulose membranes. After being blocked in 5% nonfat milk for 2 hours at room temperature, the membranes were treated with anti‐CaM (1:1000, ab45689, Abcam, MA, USA), anti‐MLC2v (1:1000, ab79935, Abcam, MA, USA), anti‐MLC2v (phospho‐S20) (1:1000, ab2480, Abcam, MA, USA), anti‐cMLCK (ARM‐Mylk3 (150‐164), 1:50, generated in our lab),^11^ anti‐cMyBP‐C3 (M‐190) (1:200, sc‐67353, Santa Cruz Bio., Inc., CA, USA), anti‐cMyBP‐C‐Ser 282 (1:1000, ALX‐215‐057, Enzo Life Sci., USA), anti‐CaMKIIδ (T287) (1:200, sc‐5392, Santa Cruz Bio., Inc., CA, USA), anti‐CaMKIIδ‐p (pT287) (1:200, sc‐5392p, Santa Cruz Bio., Inc., CA, USA) and anti‐Connexin43 (1:200, sc‐13558, Santa cruz Bio., Inc., CA, USA) at 4°C overnight. The membranes were then washed and incubated with secondary antibody (1:10 000) for 1 hour at room temperature. Protein loading was confirmed using GAPDH (1:5000, G8795, Sigma, Saint Louis, MO, USA) as an internal control. Blots were detected with the Odyssey infrared imaging system (Licor, USA). Western blot bands were quantified using Quantity One software to measure band intensity (area × OD) and normalized to GAPDH band intensity. The final results were expressed as fold changes compared with the control values.

### 2.10 Data analysis

Data were calculated as mean* ±* SEM (standard error of the means) except the length of sarcomeres as mean *±* SD (standard deviation). The ANOVA test was performed for statistical comparisons among multiple groups. Differences were considered statistically significant at *P <* .05. SPSS13.0 was used for all statistical analyses.

## 3 RESULTS

### 3.1 LBPs reduced the expression of myocardial miR‐1 in vitro and in vivo

**Figure 1:**
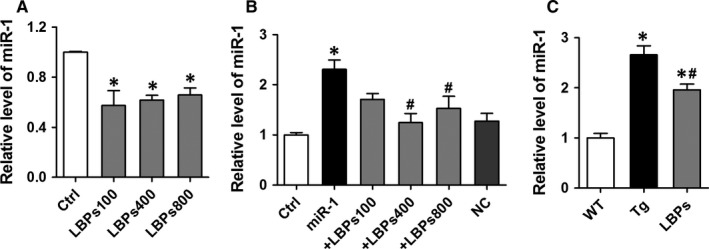
*Lycium barbarum* polysaccharides (LBPs) down‐regulated the expression level of miR‐1 in vitro and in vivo. A, The expression levels of endogenic miR‐1 in the treatment of LBPs in neonatal rat ventricular cardiomyocytes (NRVCs). NRVCs, neonatal rat ventricular cardiomyocytes; Ctrl, control. *P < .05 vs Ctrl, mean* ±* SEM, n* =* 3 independent RNA samples for each group. B, The effect of LBPs on the expression levels of miR‐1 in NRVCs overexpression model of miR‐1. *P < .05 vs Ctrl or NC, ^#^ *P <* .05 vs miR‐1; mean* ±* SEM, n* =* 8 for Ctrl, +LBPs100, +LBPs400, NC, 7 for miR‐1, and 6 for +LBPs800. C, The effect of LBPs on the expression levels of miR‐1 in miR‐1 Tg mice. Tg, transgenic; WT, wild type. *P < .05 vs WT, ^#^ *P* < .05 vs Tg; mean *±* SEM, n* =* 6 for WT, 8 for Tg, and 5 for LBPs

We quantified the miR‐1 levels in neonatal rat ventricular cardiomyocytes (NRVCs) with incremental doses of LBPs. Excitingly, our data from qRT‐PCR showed that LBPs significantly reduced the expression of endogenic miR‐1 (Figure 1A). Similar results were observed in the overexpression model of miR‐1 by transfecting miRNA mimics in NRVCs (Figure 1B). Consistently, in vivo study with miR‐1 Tg mice also confirmed the down‐regulated expression of miR‐1 in the application of LBPs (1.96 *±* 0.11 vs 2.66 *±* 0.18, *P <* .05) even not to WT level (1.96 *±* 0.11 vs 1.0 *±* 0.09, *P <* .05) (Figure 1C).

### 3.2 LBPs restored cardiac dysfunction induced by overexpression of miR‐1

**Figure 2:**
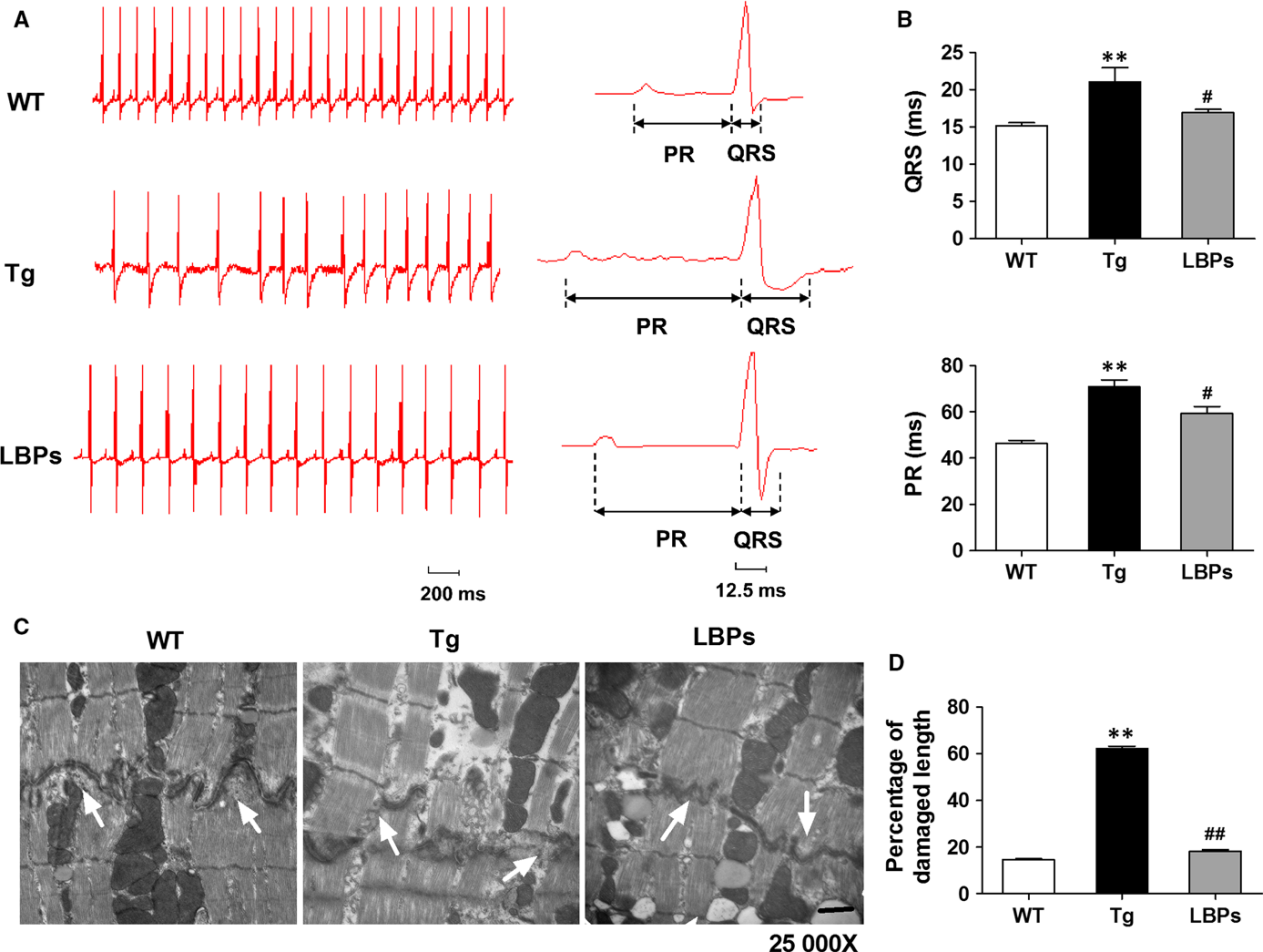
The protective effect of *Lycium barbarum* polysaccharides (LBPs) on impaired cardiac conduction function induced by miR‐1 overexpression. A, Representative surface ECG recordings showing QRS complex and P‐R interval and arrhythmias in three groups. B, Quantitative comparison of QRS and PR interval in all groups. *P < .05, **P < .01 vs WT, ^#^ *P* < .05 vs Tg; mean *±* SEM, n* =* 15 for WT, 9 for Tg, and 7 for LBPs. C, Representative intercalated disc organization showing gap junction and macula adherents detected by TEM. Magnification ×25 000, scale bar: 1 μm. D, Quantitative analysis of percentage of damaged length in intercalated disc. **P < .01 vs WT, ^##^ *P <* .01 vs Tg, data presented as mean *±* SEM, n* =* 3 independent TEM images for each group

ECG analysis illustrated significantly widened QRS complex (21.03 *±* 1.94 vs 15.17 *±* 0.38, *P <* .01) and prolonged P‐R interval (70.78 *±* 3.07 vs 46.36 *±* 1.48, *P <* .01) in Tg mice, indicating slowing down of cardiac conduction. Representative ECG tracing also recorded arrhythmias in Tg mice. Treatment with LBPs significantly improved the abnormal QRS complex and P‐R interval (16.88 *±* 0.47 vs 21.03 *±* 1.94 and 59.37 *±* 3.01 vs 70.78 *±* 3.07, respectively, *P <* .05) with no arrhythmias (Figure 2A,B). TEM examination found that intercalated discs were dissolved markedly with vacuolar degeneration of gap junctions and decreased density of the macula adherents in the hearts of Tg mice but not in those treated with LBPs (Figure 2C,D). Figure 2D presented the quantitative analysis of percentage of damaged length in intercalated disc organization.

**Figure 3:**
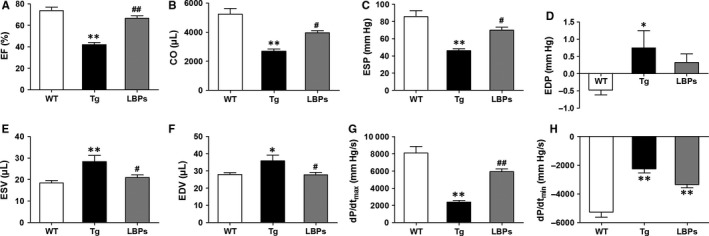
The mitigating effect of *Lycium barbarum* polysaccharides (LBPs) on damaged cardiac contractile and diastolic functions by miR‐1 overexpression. A‐H, Changes of parameters of cardiac systolic and diastolic functions in three groups. *P < .05, **P < .01 vs WT, ^#^ *P <* .05, ^##^ *P <* .01 vs Tg; mean *±* SEM, n* =* 8 for WT, 6 for Tg, and 6 for LBPs. CO, cardiac output; dP/dt_max_, maximum derivative of change in systolic pressure over time; dP/dt_min_, maximum derivative of change in diastolic pressure over time; EDP, end‐diastolic pressure; EDV, end‐diastolic volume; EF, ejection fraction; ESP, end‐systolic pressure; ESV, end‐systolic volume

P‐V loop detection revealed the changes of indexes of cardiac function. Compared to WT mice, ejection fraction (EF), the most important parameter representing contractile function, was significantly reduced by 42.99% in Tg mice (42.08 *±* 2.00 vs 73.81 *±* 3.25, *P <* .01). However, in LBPs‐treated mice EF showed significant mitigation (66.65 *±* 2.41 vs 42.08 *±* 2.00, *P <* .01) which almost moved back to normal level (Figure 3A). In addition, the impaired cardiac contractile and diastolic functions of Tg mice were indicated by decreased CO, ESP, dP/dt_max_ and dP/dt_min_, as well as increased EDP, ESV and EDV (Figure 3B‐H). Interestingly, in the treatment of LBPs, the indexes of cardiac contractile function including CO, ESP, ESV and dp/dt_max_ were seen significant restorations. However, except EDV, the indexes of cardiac diastolic function including EDP and dP/dt_min_ were not improved significantly in mice treated with LBPs (Figure 3B‐H). These implied the beneficial effect of LBPs on contractile function and at least partial recovery on diastolic function.

### 3.3 LBPs recovered adverse cardiac structural remodelling in miR‐1 Tg mice

**Figure 4:**
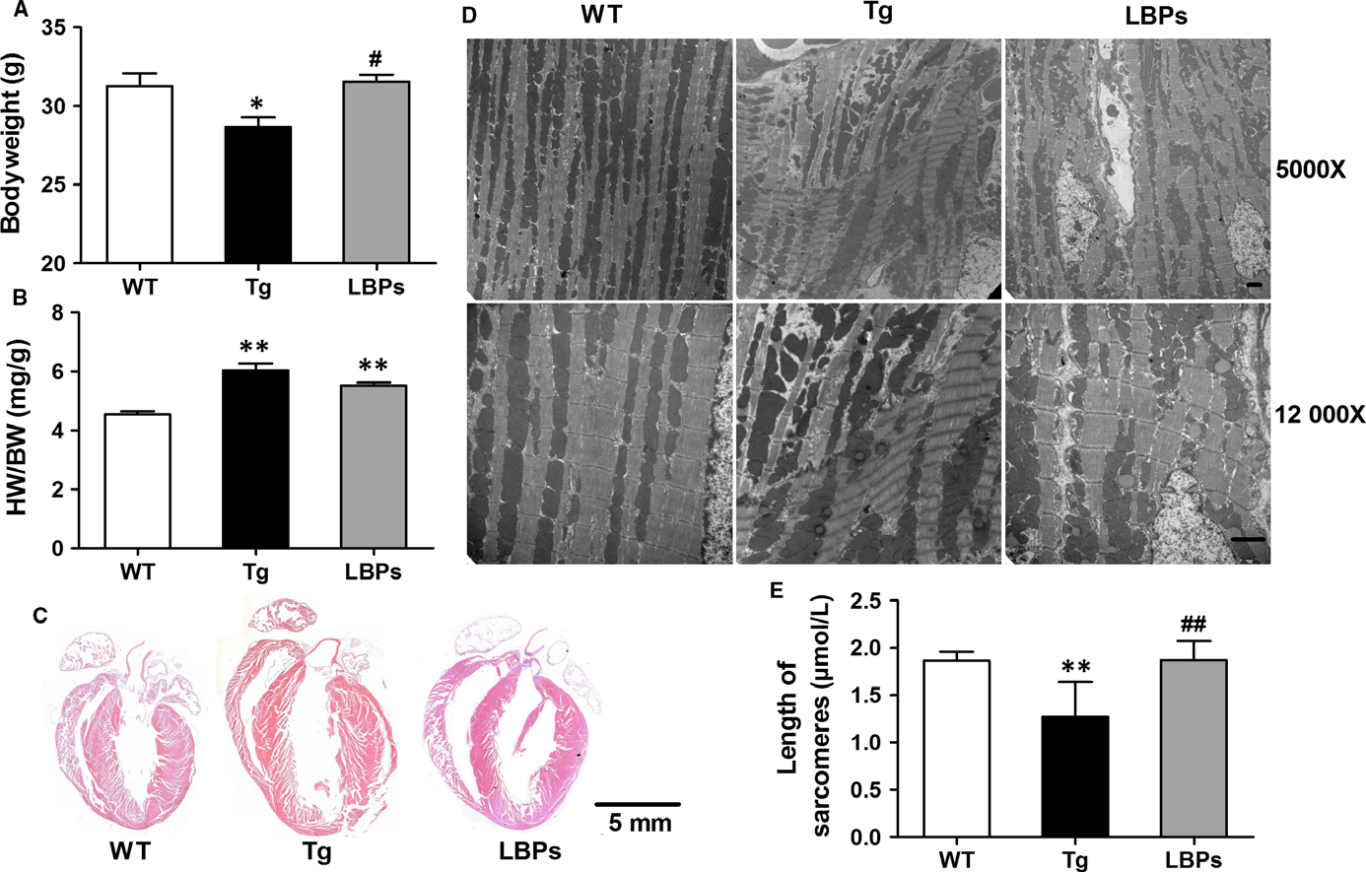
The reversing effect of *Lycium barbarum* polysaccharides (LBPs) on cardiac structural remodelling produced by miR‐1 overexpression. A, The change of bodyweight in three groups. *P < .05 vs WT, ^#^ *P <* .05 vs Tg; mean *±* SEM, n* =* 13 for WT, 12 for Tg, and 7 for LBPs. B, The change of ratio of heart to bodyweight in three groups. **P < .01 vs WT; mean* ±* SEM, n* =* 13 for WT, 12 for Tg, and 7 for LBPs. C, Longitudinal sections of hearts stained by H&E from mice of three groups, scale bar: 5 mm. D, TEM examination of cardiac myofilament showing sarcomeric length, clear zone and H‐zone, and myofibrillar fragmentation and dissolution (in Tg mice). Up panels: magnification ×5000, scale bar: 2 μm and down panels: magnification ×12 000, scale bar: 2 μm. E, Comparison of length and variation of sarcomeres. **P < .01 vs WT, ^##^ *P <* .01 vs Tg, data presented as mean *±* SD (for variation), n* =* 80 from three hearts for each group

Decreases in bodyweight and increases in heart‐to‐bodyweight ratios (HW/BW) were obtained from Tg mice as compared to WT ones. In the treatment of LBPs, significant reversion was observed in bodyweight but not in HW/BW (Figure 4A,B). H&E staining exhibited that the hearts of Tg mice were enlarged in the diastolic state and LBPs‐treated ones were not recovered to normal (Figure 4C), which was consistent with the result of HW/BW. TEM examination displayed shortened and uneven sarcomeric length (asynchronous contraction) as well as the loss of clear zone and H‐zone in Tg mouse hearts, and even severe myofibrillar fragmentation and dissolution of cardiomyocytes (Figure 4D,E), suggesting that remodelled sarcomeric assembly participated in the detrimental alterations of heart function in Tg mice. But remarkably, these phenomena were not found in mice treated with LBPs, indicating the protective effect of LBPs on adverse structural remodelling and consequent heart dysfunction (Figure 4D,E). Figure 4E presents a summary of length of sarcomeres with error bar (standard deviation) expressing the variation of sarcomeres.

### 3.4 LBPs reversed the reductions of target proteins of miR‐1 and key contractile proteins

**Figure 5:**
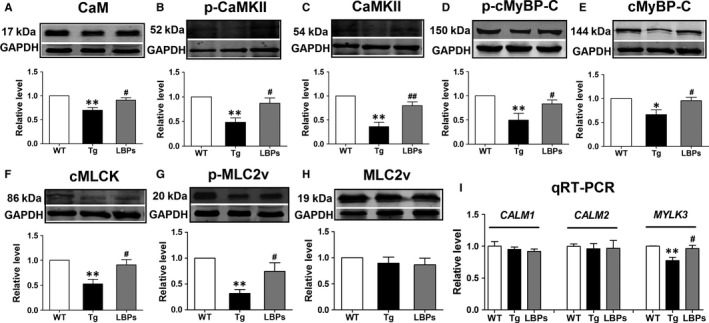
The restoration of *Lycium barbarum* polysaccharides (LBPs) on reduced expression of key cardiac contractile regulatory proteins in miR‐1 Tg mice. A‐H, Representative examples of Western blot bands of each protein and statistical bar graph indicating the result of densitometric analysis of the bands as normalized to the quantity of GADPH protein. *P < .05, **P < .01 vs WT, ^#^ *P <* .05, ^##^ *P <* .01 vs Tg; mean *±* SEM, n* =* 5, 6, 6, 4, 5, 5, 7, 6 independent protein samples for each group in sequence from A to H. CaM, calmodulin; CaMKII, Ca^2+^/calmodulin‐dependent protein kinase II; cMyBP‐C, cardiac myosin binding protein C; cMLCK, cardiac myosin light chain kinase; MLC2v, myosin light chain 2v. I, Comparison of mRNA levels of *CALM1*,*CALM2* and *MYLK3* by qRT‐PCR in all groups. **P < .01 vs WT, ^#^ *P <* .05 vs Tg; mean *±* SEM, n* =* 5 independent RNA samples for each group

In the present study, the repressive effects of miR‐1 on the target proteins calmodulin (CaM) and cardiac myosin light chain kinase (cMLCK) are shown in Figure 5A,F as observed by a weaker signal vs WT band. Administration of LBPs displayed more intense band indicating increased expression of target proteins. Similarly, both the total and phosphorylated protein levels of Ca^2+^/calmodulin‐dependent protein kinase II (CaMKII) were decreased in Tg hearts as compared to those from WT, and consistently, the reversion were observed in LBPs treated ones (Figure 5B,C). Similar trends were also obtained in cardiac myosin binding protein C (cMyBP‐C) (Figure 5D,E). The reduction in phosphorylation status of myosin light chain 2v (MLC2v) in hearts from Tg mice was significantly recovered in those from LBPs‐treated mice (Figure 5G), although the expression of the total protein level of MLC2v was in the same range in three groups (Figure 5H). Furthermore, *MYLK3* (encoding cMLCK) mRNA level was also decreased in Tg mice and reversed by LBPs treatment, but mRNA levels of *CALM1* and *CALM2,* both encoding CaM, were not changed in three groups (Figure 5I). These results indicated that the observed decrease in CaM protein expression may be ascribed at least partially to the post‐transcriptional regulation.

## 4 DISCUSSION

MiRNA‐based therapy has been recognized to be a promising novel therapeutic strategy for the treatment of cardiovascular diseases.^29^, ^30^ The principal finding of this study is the first to identify that LBPs protect the hearts from adverse structural remodelling and impaired cardiac function in miR‐1 transgenic (Tg) mouse line. The mechanism of cardioprotective effects of LBPs is due, at least partially, to down‐regulating the expression of miR‐1 to reverse miR‐1‐induced repression of target proteins relevant to myocardial contractibility. LBPs could serve as a new candidate for the treatment of HF.

At present, the conventional treatments for HF are mainly angiotensin converting enzyme inhibitors, β‐adrenoceptor blockers and diuretics. The important effectiveness, especially in short‐term use, cannot be denied, but long‐term use does not prolong the life‐span of those patients with many adverse side‐effects such as producing poor compliance, promoting myocardial injury and remodelling, deteriorating cardiac function and even increasing the rate of sudden death caused by cardiac arrhythmia.^1^, ^31^ Treatment of HF remains unsatisfactory as available treatments often fail to control the symptoms.^32^ Therefore, the current major goal for HF is still to develop rational approaches to improve the quality of life and prolong life‐span. The use of natural compounds to improve human health has long been recognized in the history and increased in popularity in the modern society. The traditional Chinese herb, compared to the Western medicine, has a prominent advantage because of a stable curative effect with significantly less toxicity. With the progression of modern technology, pharmaceutics of herbal medicine products is undergoing rapid development in China, and more and more herbal compound extracts are being authenticated, standardized and administered successfully in clinical practice.^33^, ^34^ Traditional Chinese medicine Tongxinluo (TXL) mediates endothelial preservation by mitigating atherogenesis^35^ and improves cardiac functions in response to ischaemia–reperfusion injury.^36^ Qili qiangxin capsule, a traditional Chinese medicine, has been approved in China to be used in combination therapy for chronic HF.^34^ Of note, several other traditional Chinese medicines such as Huangqi injection, Berberine, Shenfu decoction, Shengmai, Hawthorn, Curcumin, Resveratrol and Cannabinoids may have potential therapeutic use in HF as adjunctive treatments in clinical practice or animal models of human disease.^31^, ^37^, ^38^, ^39^, ^40^ All these findings manifest that herbal medicine products play more and more important role in modern medical therapy.

*Lycium barbarum* (also known as Goji berry or wolfberry), a famous Chinese medicinal herb and also a functional food, has a long history of use in a broad spectrum of diseases to nourish liver, kidneys and eyes and becomes increasingly popular in Europe and North America.^19^, ^41^, ^42^ Polysaccharides are the most important functional ingredient that is approximately 40% of dry mass in *L. barbarum* fruits.^18^ Till recent several years LBPs are given increasing attention in the field and most researches focus on its anti‐oxidative property as a free radical scavenger for immunomodulation and anticancer.^14^, ^15^, ^16^ The studies of LBPs on cardiovascular aspect are only limited to the protective phenomena (no discussion of mechanism) on doxorubicin‐induced cardiotoxicity and ischaemia/reperfusion injury of rat heart.^21^, ^22^, ^23^, ^24^ In the present study, we used the model of miR‐1 Tg mice and demonstrated that LBPs restored the cardiac function impaired by overexpression of miR‐1, as indicated by increase in EF, CO, ESP, and dp/dt_max_, as well as decrease in ESV and EDV. Notably, although the indexes of cardiac diastolic function EDP and dP/dt_min_ were not improved significantly, TEM results of administration of LBPs did not show morphological damages on sarcomeric assembly, such as shortened and uneven sarcomeric length, loss of clear zone and H‐zone and even severe myofibrillar fragmentation and dissolution as displayed in Tg mouse hearts. Additionally, the treatment with LBPs displayed normal intercalated disc and gap junction in TEM and reversed conduction abnormalities induced by miR‐1 in ECG, strongly implying the protective effect of LBPs on cardiac conduction function. Therefore, our study has uncovered for the first time that LBPs possess the protective action against the structural and functional abnormalities of the heart, the essential aetiology of HF.

**Figure 6:**
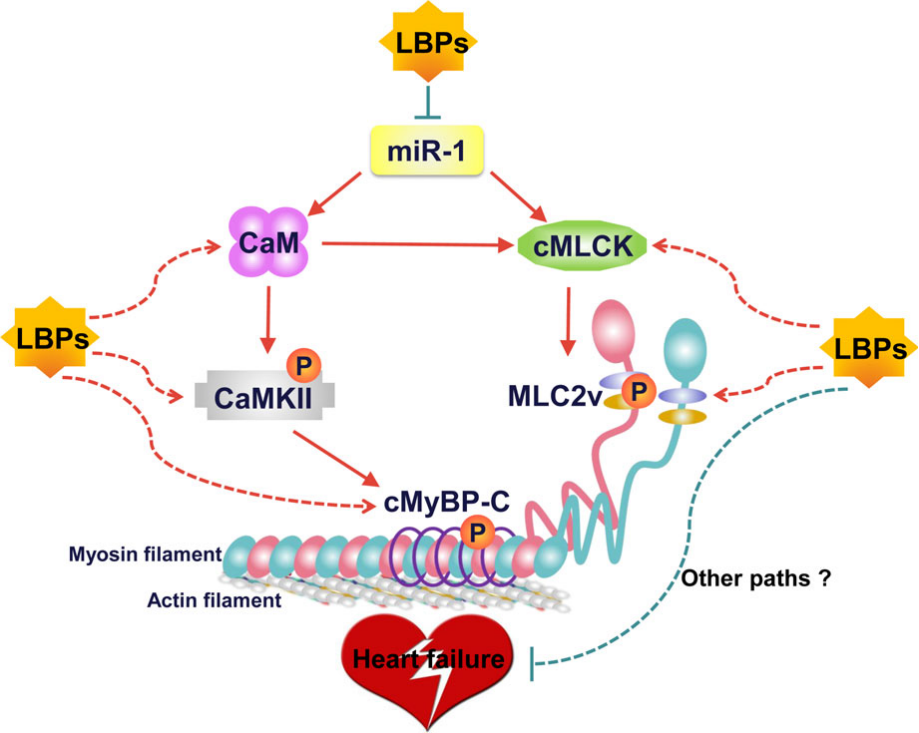
Schematic illustration explaining the possible targeting and signalling mechanisms by which *Lycium barbarum* polysaccharides (LBPs) restore the impairment of cardiac contractility induced by miR‐1 overexpression. LBPs restored the reductions of CaM and cMLCK, target proteins affected by miR‐1, and their corresponding downstream proteins for myocardial contraction including CaMKII, cMyBP‐C and MLC2v. LBPs might perform cardioprotective effects by affecting key contractile proteins directly or other signalling pathway. CaM: calmodulin; cMLCK, cardiac specific myosin light chain kinase; CaMKII, calmodulin‐dependent protein kinase II; cMyBP‐C, myosin binding protein‐C; MLC2v, myosin regulatory light chain 2

HF with complicated pathogenesis is associated with loss of cardiac contractility, abnormalities in Ca^2+^ handling and altered phosphorylation states of cardiac contractile regulatory protein.^43^, ^44^ Our previous study has elucidated that overexpression of miR‐1 repressed potential target proteins CaM and cMLCK, which attenuated the phosphorylation of CaMKII, cMyBP‐C and MLC2v, leading to impaired sarcomeric assembly and consequent heart dysfunction.^11^ CaM, a known transducer of Ca^2+^ signal, activates CaMKII^45^ to directly phosphorylate cMyBP‐C, which is a thick filament protein with physiological significance for normal myocardial contractility and stability and serves as a convergent node for signalling processes in the cardiomyocyte.^46^, ^47^ Activation of cMLCK, also regulated by CaM, appears to be pivotal to maintain the phosphorylation of MLC2v, which functions as an essential component of thick myofilament assembly and plays a critical role in maintaining normal myocardial contractility and function.^48^, ^49^ The dephosphorylation of both MLC2v and cMyBP‐C is associated with a declined cardiac function in failing human hearts and animal models of HF.^46^, ^48^ The present study exhibited that LBPs restored the reductions in phosphorylation of MLC2v and both total level and phosphorylation of CaMKII and cMyBP‐C in Tg mice, as well as their upstream activators cMLCK and CaM, target proteins affected by miR‐1. Undoubtedly all these data supported that the structural and functional protection of LBPs on Tg mouse hearts was due largely to the down‐regulation of miR‐1 level and subsequent restoration of target proteins essential for cardiac contractile function. Other studies found that tanshinone IIA, an active component of a traditional Chinese medicine based on Salvia Miltiorrhiza, protected against arrhythmogenesis after myocardial infarction and cardiac sudden death induced by lethal arrhythmias via repression of miR‐1,^8^, ^13^ and Propranolol exerted ischaemic cardioprotection related to down‐regulation of miR‐1,^7^ providing powerful supports for our cardioprotective result of LBPs on HF by down‐regulation of miR‐1. These findings not only help us understand the mechanisms underlying the beneficial effects of LBPs on HF, but also conceptually advance our view regarding miRNAs to serve as potential therapeutic drug targets. Notably, although the suppression did not completely back to normal levels as indicated in WT mice, the remarkable mitigation of LBPs on adverse structural remodelling, cardiac contractile function and key contractile regulatory proteins, revealed that, besides down‐regulation of miR‐1 expression, LBPs might perform cardioprotective effects by directly affecting key contractile proteins or other signalling pathway which remains to be illuminated in future study (Figure 6).

It is undeniable that our animal model cannot represent totally as a conventional HF model. Usually in laboratory research, there are two main HF models including transaortic constriction (TAC) and permanent ligation of the left anterior descending coronary artery (LAD) for 8 weeks.^50^, ^51^ A limitation of the study is the use of miR‐1 Tg mice. In our study, in view of the important role of miR‐1, we focus on the effect of miR‐1 on HF by the animal model of miR‐1 Tg mice and try to develop new candidates regulating miR‐1 by which to treat HF. We will verify our results in conventional HF models in future. The other limitation is that we only observed the changes of structural and functional alterations in ventricles but not in atria, which merit future studies to exploit these possibilities and better interpret the mechanisms of action of miR‐1 on heart diseases.

Systematic characterization of functional compounds in medicinal herbs and their mechanisms of action are important for providing the rationale for their efficacy and developing modern evidence‐based medicine. While *L. barbarum* fruit is widely used in China, there is still a lack of in‐depth study on the pharmacological effects of its active ingredients, especially those that exert cardioprotection. Our research addressed the protection of LBPs on cardiac contraction and conduction dysfunction and adverse structural remodelling induced by overexpression of miR‐1 via direct and indirect improvement of key proteins essential for cardiac contractile function, and provided new insights into the significant role of LBPs as a new, at least a very useful adjunctive, candidate for prevention and therapy of HF. Herein, we have accumulated scientific evidence for LBPs’ cardioprotective role and hope that these novel findings will develop its potential as an evidence‐based medicine and expand the application of LBPs on heart disease.

## CONFLICT OF INTEREST

The authors declare no conflict of interest.
